# Vildagliptin ameliorates pulmonary fibrosis in lipopolysaccharide-induced lung injury by inhibiting endothelial-to-mesenchymal transition

**DOI:** 10.1186/s12931-017-0660-4

**Published:** 2017-10-16

**Authors:** Toshio Suzuki, Yuji Tada, Santhi Gladson, Rintaro Nishimura, Iwao Shimomura, Satoshi Karasawa, Koichiro Tatsumi, James West

**Affiliations:** 1 Department of Medicine, Division of Allergy, Pulmonary, and Critical Care Medicine, Vanderbilt University Medical Center, Nashville, TN 37232 USA; 2 Department of Respirology, Graduate School of Medicine, Chiba University, Chiba, Japan; 3 Department of Advanced Medicine in Pulmonary Hypertension, Graduate School of Medicine, Chiba University, Chiba, Japan; 4 Department of Emergency and Critical Care Medicine, Graduate School of Medicine, Chiba University, Chiba, Japan

**Keywords:** Endothelial-to-mesenchymal transition, Pulmonary fibrosis, Dipeptidyl peptidase 4, Post ARDS pulmonary fibrosis

## Abstract

**Background:**

Pulmonary fibrosis is a late manifestation of acute respiratory distress syndrome (ARDS). Sepsis is a major cause of ARDS, and its pathogenesis includes endotoxin-induced vascular injury. Recently, endothelial-to-mesenchymal transition (EndMT) was shown to play an important role in pulmonary fibrosis. On the other hand, dipeptidyl peptidase (DPP)-4 was reported to improve vascular dysfunction in an experimental sepsis model, although whether DPP-4 affects EndMT and fibrosis initiation during lipopolysaccharide (LPS)-induced lung injury is unclear. The aim of this study was to investigate the anti-EndMT effects of the DPP-4 inhibitor vildagliptin in pulmonary fibrosis after systemic endotoxemic injury.

**Methods:**

A septic lung injury model was established by intraperitoneal injection of lipopolysaccharide (LPS) in eight-week-old male mice (5 mg/kg for five consecutive days). The mice were then treated with vehicle or vildagliptin (intraperitoneally, 10 mg/kg, once daily for 14 consecutive days from 1 day before the first administration of LPS.). Flow cytometry, immunohistochemical staining, and quantitative polymerase chain reaction (qPCR) analysis was used to assess cell dynamics and EndMT function in lung samples from the mice.

**Results:**

Lung tissue samples from treated mice revealed obvious inflammatory reactions and typical interstitial fibrosis 2 days and 28 days after LPS challenge. Quantitative flow cytometric analysis showed that the number of pulmonary vascular endothelial cells (PVECs) expressing alpha-smooth muscle actin (α-SMA) or S100 calcium-binding protein A4 (S100A4) increased 28 days after LPS challenge. Similar increases in expression were also confirmed by qPCR of mRNA from isolated PVECs. EndMT cells had higher proliferative activity and migration activity than mesenchymal cells. All of these changes were alleviated by intraperitoneal injection of vildagliptin. Interestingly, vildagliptin and linagliptin significantly attenuated EndMT in the absence of immune cells or GLP-1.

**Conclusions:**

Inhibiting DPP-4 signaling by vildagliptin could ameliorate pulmonary fibrosis by downregulating EndMT in systemic LPS-induced lung injury.

**Electronic supplementary material:**

The online version of this article (10.1186/s12931-017-0660-4) contains supplementary material, which is available to authorized users.

## Background

The morbidity and mortality of acute respiratory distress syndrome (ARDS) is especially high when it leads to persistent intra-alveolar and interstitial fibrosis [1, 2]. The most common cause of ARDS is sepsis that involves direct or indirect interactions between endotoxins and pulmonary vascular endothelial cells (PVECs).

Our recent study showed that one of the innate survival strategies for non-apoptotic injured PVECs is endothelial-to-mesenchymal transition (EndMT) [3], which is a process wherein endothelial cells lose an endothelial cell phenotype and acquire a mesenchymal cell phenotype [4]. There are many pathways, such as those that include the transforming growth factor β (TGFβ) superfamily, through which EndMT is induced [4, 5].

We recently reported that transient EndMT was observed in mice with septic acute lung injury (ALI) that can be repaired [3]. LPS-induced EndMT was reported to be dependent on ROS levels in vitro [6], and in vivo [3]. In the repairable ALI mouse model, transient EndMT cells have a progenitor cell-like phenotype that could be involved in endothelial repair after pulmonary vascular injury [3]. Some of pulmonary microvascular endothelial cells transform into mesenchymal phenotype with retaining reversibility to their original phenotype when endotoxin stimulation is transient. However, it is unclear whether continuous or intermittent endotoxin exposure promotes complete EndMT and pulmonary fibrosis.

Indeed, numerous studies found that endothelial cells are one source of fibroblasts/myofibroblasts in fibrotic diseases [7–9]. The etiology of fibrotic diseases can vary widely. Given that one of the main initial targets of endotoxins is vascular endothelial cells especially in case of ARDS from extra-pulmonary origin [6, 10], EndMT could thus be closely involved in the pathogenesis of pulmonary fibrosis after systemic endotoxemic injury.

CD26/dipeptidyl peptidase 4 (DPP-4) is broadly expressed by a variety of cell types in the lung, including capillary endothelial cells [11]. DPP-4 inhibitors are widely used glucose-lowering drug that inhibit the breakdown of the incretin hormone glucagon-like peptide-1 (GLP-1) [12]. They have recently gathered increasing interest since they might have beneficial effects on cardiovascular diseases [13–15]. Although most of the previous reports explained their vascular protective effects by the upregulation of GLP-1 and lowering blood glucose level [16, 17], it was also reported that they could act in some part not through the breakdown of GLP-1. Kohashi et al. elegantly revealed that DPP-4 inhibitor but not GLP-1 reduced incidence of angiotensin II-induced abdominal aortic aneurysm in *ApoE* ^−*/*−^ mice [18]. In addition, DPP-4 inhibition was reported to prevent systemic inflammation, vascular dysfunction and end organ damage in endotoxemic conditions [19], and ameliorate kidney fibrosis in diabetic mice [20]. Thus, it has been revealed that DPP-4 inhibitors have various effects without increasing insulin secreting property from pancreas β-cell or GLP-1.

Based on these findings, we hypothesize that EndMT is involved in the pathogenesis of pulmonary fibrosis after systemic endotoxemic injury, which can be attenuated by the DPP-4 inhibitor vildagliptin. In the present study, we examined whether intermittent LPS exposure leads to pulmonary fibrosis via EndMT. We also evaluated the therapeutic potential of vildagliptin for inhibiting post-ALI pulmonary fibrosis partly via GLP-1-independent antioxidant pathway.

## Methods

### Mouse model of pulmonary fibrosis after systemic endotoxemic injury

We modified the experimental protocol for induction of pulmonary fibrosis in mice by intermittent intraperitoneal lipopolysaccharide (LPS) injection [21]. Seven- to eight-week-old male C57BL/6 mice (Clea Japan, Tokyo, Japan) received intraperitoneal administration of 5 mg/kg body weight LPS for five consecutive days. The LPS was derived from *Escherichia coli* (O55:B5 Sigma, St. Louis, MO) and dissolved in PBS. For treatment in vivo with vildagliptin, mice were given once daily doses of either 10 mg/kg vildagliptin (Santa Cruz Biotechnology, Dallas, TX) or saline vehicle delivered by intraperitoneal injection for 14 consecutive days from 1 day before the first administration of LPS. At 14 days and 28 days after starting LPS injection, mice were anaesthetized, and lung tissues were quickly removed and processed as described below. All animal experiments were conducted under protocols approved by the Chiba University Institutional Review Board for animal experiments.

### Lung histological analyses

Resected lungs were formalin fixed and embedded in paraffin. Lung sections (2 μm) were deparaffinized in xylene, hydrated using ethanol, and stained with Elastica van Gieson (EVG) stain using standard protocols for morphological analyses. The pulmonary fibrosis severity was semi-quantitatively assessed according to the method proposed by Ashcroft [22].

### Fluorescent immunohistochemistry

Lungs were embedded in Tissue-Tek^®^,O.C.T. Compound (SAKURA Finetek, Tokyo) and frozen in liquid nitrogen for preparation of cryosections. Frozen lung tissues were cut into 6 μm thick sections, immunostained and visualized by confocal microscopy (Fluoview FV 10i, Olympus, Tokyo). The sections were fixed in acetone for 10 min, blocked with Block Ace (Dainippon Sumitomo Pharma, Tokyo) for 10 min, and incubated with the primary and secondary antibodies for 60–120 min. The following antibodies were used for immunostaining: anti-CD31-Alexa488 (BioLegend, San Diego, CA), anti-CD45-Alexa647 (BioLegend), anti-CD26 (R&D Systems, Minneapolis, MN), anti-α-SMA (Thermo Scientific, Waltham, MA), and anti-S100 calcium-binding protein A4 (S100A4) (Abcam, Cambridge, UK).

### Human pulmonary vascular endothelial cell injury model

Human lung microvascular endothelial cells (HMVEC-L) were purchased from Clonetics (Walkersville, MD) and cultured in endothelial cell basal medium-2 (EBM-2, Lonza, Walkersville, MD) supplemented with 10% fetal bovine serum and endothelial cell growth medium 2 (EGM-2 SingleQuots, Invitrogen, Carlsbad, CA). All cells were maintained at 37 °C in a 5% CO_2_ humidified incubator. Cells were cultured to 90% confluence and transitioned to starvation medium that included EBM-2 supplemented with 1% fetal bovine serum, 0.1% gentamicin sulfate and amphotericin-B, heparin, and ascorbic acid for 24 h. Cells were exposed to vehicle (PBS) or LPS (20 μg/ml) with or without DPP-4 inhibitor (Vildagliptin 10 nM. Linagliptin 100 nM.) in fresh starvation medium at 37 °C for 96 h.

### Single cell suspension

At the time of harvest, mouse lungs were perfused with 30 ml PBS containing 10 U/ml heparin (Novo-Heparin, Mochida, Tokyo) from the right ventricle until there was no visible blood. The tissue was then minced and placed in an enzyme cocktail consisting of DMEM (Sigma), 1% BSA (Wako, Osaka, Japan), 2 mg/ml collagenase (Wako), 100 μg/ml DNase (Wako), and 2.5 mg Dispase II (Roche Diagnostics GmbH, Mannheim, Germany) at 37 °C for 60 min before the tissue was passed through a nylon cell strainer with a 100 μm mesh size.

### Flow cytometry (FCM) of lung cells

Mouse lung cells were pretreated with anti-CD16/32 antibody (BioLegend) to block Fc receptors, and then incubated with specific antibodies at 4 °C in the dark. The following antibodies were used for cell surface staining: anti-CD31-PE/Cy7 (BioLegend), anti-CD45-Alexa700 (BioLegend) and anti-CD26-PE (BioLegend). To measure α-SMA and S100A4 levels, after surface staining the cells were incubated with anti-α-SMA (Thermo Scientific) and anti-S100A4 (Abcam) for 35 min at 22 °C. Cells were then incubated for 25 min at 22 °C with donkey anti-rabbit IgG-Alexa 488 (IgG; H + L) (Life Technologies) as a secondary antibody.

HMVEC-Ls were pretreated with anti-CD16/32 antibody and then incubated with anti-CD31, −CD45, and -α-SMA. Cell fluorescence was measured using a FACSCantoTM II instrument (Becton Dickinson, San Jose, CA) and the output was analyzed with FlowJo software (Tree Star, San Carlos, CA).

### Isolation of mouse PVECs and mouse mesenchymal cells

Mouse PVECs were defined as CD31^+^/CD45^−^/CD326^−^ cells, and mouse mesenchymal cells were defined as CD31^−^/CD45^−^/CD326^−^ cells. Each cell type was sorted using the BD FACS Aria II cell sorter as previously reported [23]. Propidium iodide (0.5 μg/ml) (Thermo Scientific) staining was used to exclude dead cells.

### Fluorescent immunocytochemistry (ICC)

Isolated mouse PVECs were fixed in a 1:1 mixture of methanol and acetone for 2 min followed by blocking with normal goat serum for 30 min as per our previous report [3]. The cells were incubated with primary antibodies (anti-α-SMA and anti-CD31) for 1 h at room temperature, and then with secondary antibodies for 1 h at room temperature. Finally, Hoechst 34,580 (Sigma) was used to identify cell nuclei, and the cells were examined by confocal microscopy (Fluoview FV 10i, Olympus). HMVEC-Ls cultured with or without LPS for 96 h were immunostained using the same method.

### qRT-PCR analysis

Total RNA from CD31^+^/CD45^−^/CD326^−^ cells and CD31^−^/CD45^−^/CD326^−^ cells were isolated with Nucleo Spin RNA XS (MACHEREY NAGEL GmbH & Co. KG, Düren, Germany) according to the manufacturer’s instructions. RNA was subjected to RT-PCR with SuperScript VILO (Life Technologies) according to the manufacturer’s protocol and single stranded cDNA was synthesized. The resulting cDNA samples were subjected to PCR for amplification using an ABI Prism 7300 Sequence Detection System (Applied Biosystems, Carlsbad, CA). Specific primers and probes were designed using Web-based software from the Universal ProbeLibrary Assay Design Center (Roche Applied Science). The Ct value for each sample was normalized with respect to *Hprt1* as an endogenous control gene and the relative expression level was calculated using the 2^-ΔΔCt^ method. The details of the primer sequences are described in Additional file 1.

### Reactive oxygen species (ROS) generation assay

After surface staining, the cells were incubated in PBS containing 40 μM of dichlorofluorescein diacetate (DCFDA; Life Technologies) for 30 min at 37 °C, to measure intracellular ROS.

### Cell migration assay

The differences in migration of cells that had a mesenchymal cell origin was evaluated using the Oris™ Cell Migration Assay (Platypus Technologies, Madison, WI) according to the manufacturer’s protocol. In brief, isolated PVECs and mesenchymal cells from mouse lungs were stimulated with 20 μM LPS, and the cells were grown to 90% confluence before removal by trypsinization, resuspension in appropriate medium, and seeding into Oris™ Pro Collagen 96-well plates with an Oris™ cell seeding stopper to restrict cell seeding to the outer regions of the wells. The plates were seeded with 300,000 cells in 100 μl of appropriate medium per well. The seeded plates were incubated for 24 h at 37 °C in 5% CO_2_ to allow cell attachment, and the stoppers were then removed to create a 2 mm diameter detection zone into which cells could migrate. After removal of the stoppers, the 96-well plates were incubated for 48 h at 37 °C in 5% CO_2_ to allow time for migration, and the number of cells that had migrated into the detection zone was determined.

### Statistical analysis

Values are shown as mean ± SEM unless otherwise described or the median (25–75th percentile). The results were analyzed using the Mann-Whitney test for comparison between any two groups, and by nonparametric equivalents of ANOVA for multiple comparisons. GraphPad PRISM software (Version 7.03; GraphPad Software, San Diego) was used for data analysis. The level of statistical significance was set at *P* < 0.05.

## Results

### Vildagliptin attenuated pulmonary fibrosis induced by LPS

We used a post-ALI pulmonary fibrosis model in which LPS treatment of male C57BL/6 mice induced pulmonary fibrosis as described in a previous report [21].

**Fig. 1:**
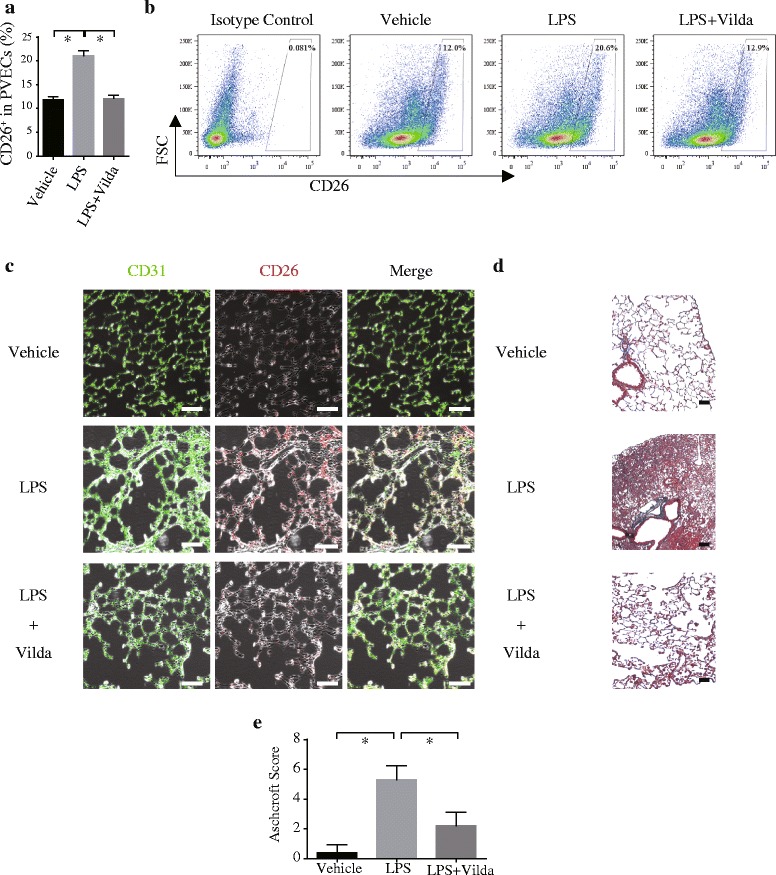
Vildagliptin restored normal pulmonary structure in a mouse model of post-ALI pulmonary fibrosis. **a** Flow cytometry (FCM) analyses revealed that the number of CD26-expressing pulmonary vascular endothelial cells (PVECs: CD31^+^/CD45^−^ cells) isolated from mice was significantly increased 14 days after LPS administration. This effect was significantly inhibited by systemic vildagliptin administration (*P < 0.05, *N* = 5). **b** Representative FCM panels with CD26^+^-gated PVECs. **c** Immunohistochemistry also revealed that the number of CD26-expressing pulmonary vascular endothelial cells (PVECs: CD31^+^CD45^−^ cells) significantly increased 14 days after LPS administration, and this increase could be significantly inhibited by systemic vildagliptin administration. CD31, green; CD26, blue; CD45, red. Scale bars, 100 μm. **d** Effect of bleomycin on lung architecture in vehicle- or vildagliptin-treated mice as shown by Masson’s trichrome staining of lung tissue sections 28 days after LPS administration. **e** The Ashcroft fibrosis score was used to compare the degrees of pulmonary fibrosis. Pulmonary fibrosis induced by LPS was significantly attenuated by vildagliptin treatment (*P < 0.05, *N* = 5). Scale bars, 100 μm.

Both flow cytometry (FCM) and immunohistochemical analysis revealed that CD26 expression in PVECs defined as CD31^+^CD45^−^ cells was increased 14 days after LPS administration (Fig. 1a-c). Furthermore, Masson’s trichrome staining of lung sections to evaluate fibrotic lesions showed that recurrent LPS exposure led to prominent pulmonary fibrosis, and this fibrosis was attenuated in the presence of the DPP-4 inhibitor vildagliptin. (Fig. 1d, e).

### Vildagliptin attenuated EndMT in PVECs

Hashimoto et al. reported that EndMT is involved in bleomycin-induced pulmonary fibrosis [7]. However, less is known about the origin of pulmonary fibrosis in systemic endotoxin-induced ALI. In the pulmonary fibrosis model after systemic endotoxemia in which pathogenesis is initiated by endothelial injury, we surmised that EndMT is closely related to fibrotic processes.

**Fig. 2:**
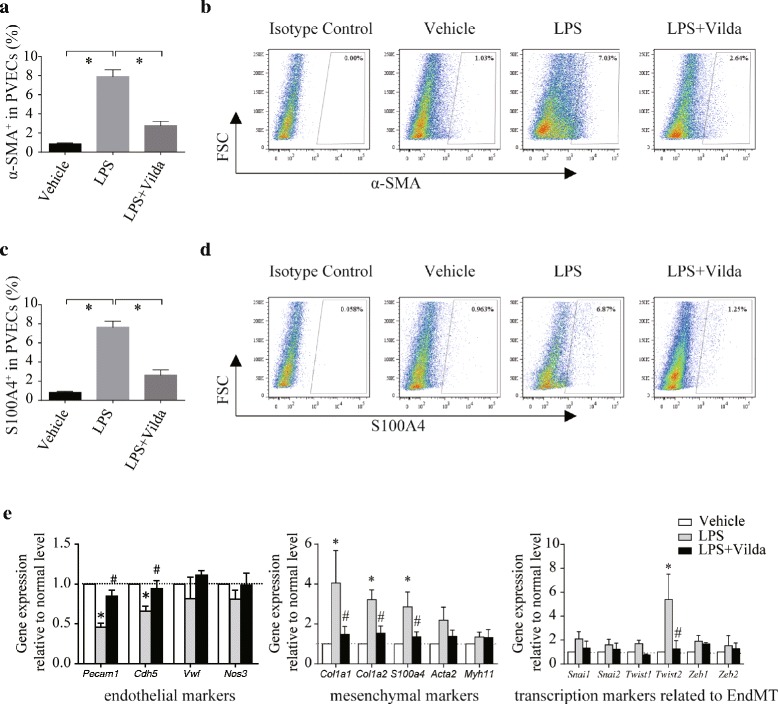
Antifibrotic effects of vildagliptin were associated with EndMT inhibition in septic lungs. **a** Flow cytometry (FCM) analyses revealed that the percentage of α-SMA^+^-gated PVECs significantly increased in LPS-induced pulmonary fibrosis, and this increase was attenuated by systemic administration of vildagliptin (*P < 0.05, N = 5). **b** Representative FCM panels with α-SMA^+^-gated PVECs. **c** FCM analyses revealed that the percentage of S100A4^+^-gated PVECs significantly increased in LPS-induced pulmonary fibrosis, and the increase was attenuated by systemic administration of vildagliptin (*P < 0.05, *N* = 5). **d** Representative FCM panels with S100A4^+^-gated PVECs. **e** Gene expression of mesenchymal-specific markers (*Col1a1*, *Col1a2* and *S100a4*) in isolated PVECs significantly increased 28 days after LPS challenge, whereas gene expression of endothelial specific markers in isolated PVECs (*Pecam1* and *Cdh5*) significantly decreased. Moreover, expression of *Twist2*, a transcription factor related to EndMT in PVECs was significantly increased 28 days after LPS challenge. All of these changes were significantly attenuated by systemic administration of vildagliptin (*P < 0.05, *N* = 5). Values are means ± SEM

We first evaluated whether EndMT is induced in a recurrent LPS ip model. FCM analyses indicated that representative mesenchymal markers, including α-SMA and S100A4, were highly co-expressed with the representative endothelial marker CD31 (Fig. 2a-d).

**Fig. 3:**
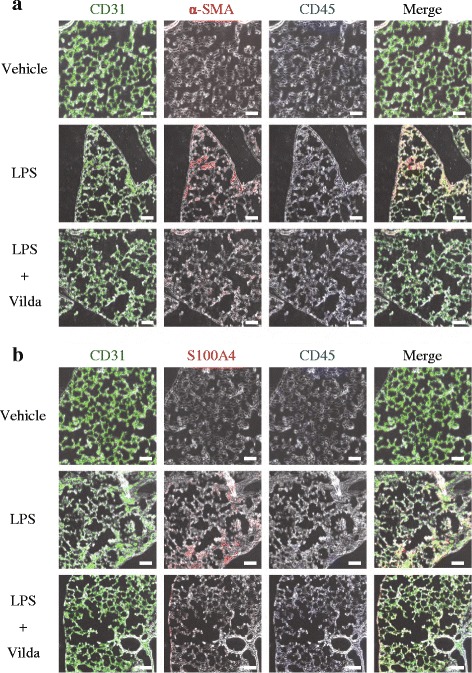
Vildagliptin suppressed EndMT in a mouse model of post-ALI pulmonary fibrosis. **a** Immunohistochemistry revealed that the number of CD31^+^/α-SMA^+^-cells (EndMT-cells) isolated from mice increased 28 days after LPS administration, and the increase could be significantly inhibited by systemic vildagliptin administration. CD31, green; α-SMA, red; Hoechst, blue. Scale bars, 100 μm. **b** Immunohistochemistry revealed that the number of CD31^+^/S100A4^+^-cells (EndMT-cells) was increased 28 days after LPS administration, and the increase could be significantly inhibited by systemic vildagliptin administration. CD31, green; S100A4, red; Hoechst, blue. Scale bars, 100 μm

We next isolated PVECs using flow cytometry and evaluated gene expression by quantitative RT-PCR analyses. Gene expression of mesenchymal-specific markers (*Col1a1*, *Col1a2* and *S100a4*) in isolated PVECs was significantly increased in LPS-treated mice compared to in vehicle-treated mice, whereas gene expression of endothelial-specific markers (*Pecam1* and *Cdh5*) in isolated PVECs was significantly decreased in LPS-treated mice (Fig. 2e). Moreover, the gene expression of *Twist 2*, one of the transcription factors related to EndMT, was significantly increased in LPS-treated mice compared to in vehicle-treated mice. Interestingly, all of these changes in expression were significantly attenuated by systemic administration of vildagliptin (Fig. 2a-e). These results were consistent with those obtained in immunofluorescence analyses using triple staining for mesenchymal cell markers (α-SMA and S100A4), CD45 and CD31 (Fig. 3a, b).

### Vildagliptin attenuated ROS production in PVECs in vivo

Since we recently reported that ROS is one of the key inducer of EndMT in septic lung injury [3], we next evaluated if vildagliptin attenuated ROS production in PVECs. As expected, intracellular ROS measured in PVECs using DCFDA significantly decreased in LPS-PVECs treated with vildagliptin (Fig. 5a).

### Vildagliptin inhibited LPS-induced EndMT of HMVEC-Ls in the absence of immune cells or GLP-1

As we recently reported, LPS directly induces pulmonary vascular EndMT in the absence of immune cells in vitro [3]. To evaluate the direct efficacy of vildagliptin in inhibiting LPS-induced EndMT in HMVEC-Ls, we next conducted in vitro experiments to test whether vildagliptin inhibited EndMT in the absence of immune cells or GLP-1.

**Fig. 4:**
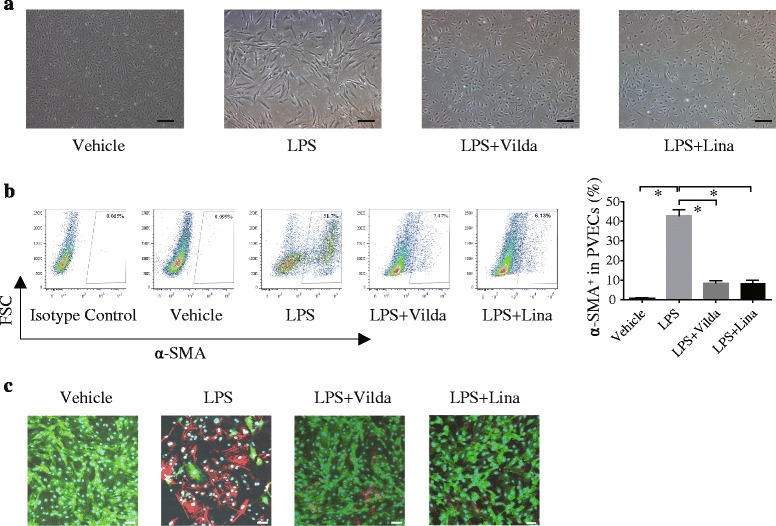
Vildagliptin inhibited LPS-induced EndMT in the absence of immune cells. **a** Phase-contrast micrographs of PVECs (CD31^+^/CD45^−^ cells) isolated from mice in the absence or presence of LPS (10 μg/ml for 144 h) and vildagliptin (10 nM)/ linagliptin (100 nM) treatment. The morphology of PVECs exposed to LPS changed to a spindle shape and vildagliptin or linagliptin treatment preserved the original morphology. Scale bars, 50 μm. **b** FCM analyses also revealed that the percentage of α-SMA^+^-PVECs significantly increased 144 h after LPS challenge and treatment by vildagliptin or linagliptin significantly suppressed this change (*P < 0.05, *N* = 5). Values are means ± SEM. **c** Immunocytochemistry revealed an increase in α-SMA^+^-PVECs 144 h after LPS challenge, and this increase was suppressed by vildagliptin or linagliptin. CD31, green; α-SMA, red; Hoechst, blue. Scale bars, 50 μm. Vilda; Vildagliptin, Lina; Linagliptin

While LPS exposure induced morphological change to a spindle-shaped phenotype, vildagliptin treatment attenuated the degree (Fig. 4a). In addition, FCM analyses revealed that vildagliptin treatment decreased upregulation of α-SMA and S100A4 expression in HMVEC-Ls (Fig. 4b). Immunocytochemistry also showed an increase in the number of α-SMA^+^-HMVEC-Ls 144 h after LPS challenge, whereas vildagliptin suppressed this increase. Since GLP-1 is produced and secreted by intestinal enteroendocrine epithelial cells, our experiments performed in vitro using a single vascular cell type provided results independent of GLP-1 participation. These data demonstrated that vildagliptin can attenuate the mesenchymal transition of endotoxin-treated PVECs partly independent of GLP-1 (Fig. 4c).

### Vildagliptin attenuated ROS production in PVECs in vitro

**Fig. 5:**
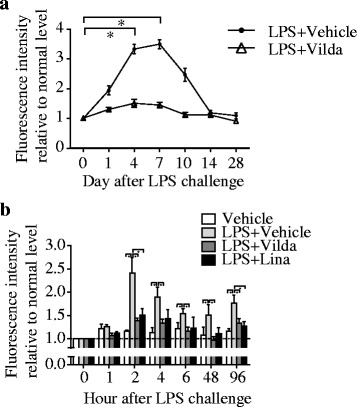
Vildagliptin attenuated ROS production in PVECs. **a** After LPS injection, intracellular ROS significantly rose in PVECs, then peaked after 7 days, and eventually returned to the base line on day 14 (*P < 0.05; *n* = 6). Intracellular ROS measured in PVECs significantly decreased in LPS-PVECs treated with vildagliptin (Vilda). PVECs ROS production was determined by FCM using DCFDA. Values are means ± SEM. **b** Fluorescence intensity of oxidized DCFDA in viable HMVEC-Ls (PI^−^/CD31^+^/CD45^−^ cells) from control- and LPS-treated HMVEC-Ls with or without vildagliptin (Vilda) or Linagliptin (Lina) are shown. The fluorescence intensity increased within 2 h of LPS challenge, which was before the increase in EndMT-HMVEC-Ls. These phenotypic changes were suppressed by vildagliptin or linagliptin (*P < 0.05; *n* = 4). Values are means ± SEM

Next, we evaluated if vildagliptin attenuated ROS production in HMVEC-Ls independent of GLP-1. Interestingly, expression of ROS was significantly decreased in LPS-HMVEC-Ls treated with vildagliptin in the absence of GLP-1 (Fig. 5b).

### Linagliptin also inhibited LPS-induced EndMT of HMVEC-Ls in the absence of immune cells

To confirm different gliptin displays the same effects to HMVEC-Ls, we used linagliptin, which had been reported to ameliorate kidney fibrosis [24]. Interestingly, linagliptin also inhibited EndMT of LPS-exposed HMVEC-Ls (Fig. 4a-c). Linagliptin also attenuated ROS production in HMVEC-Ls (Fig. 5b).

Taken together, DPP-4 inhibitors could directly inhibit EndMT partly with antioxidant pathway independent of GLP-1.

### EndMT cells had higher proliferative activity than non-endothelium-derived mesenchymal cells

Finally, to evaluate the impact of attenuated EndMT, we analyzed the cytologic characteristics of EndMT cells in PVECs (CD31^+^/CD45^−^/CD326^−^ cells) and pulmonary mesenchymal cells (CD31^−^/CD45^−^/CD326^−^ cells) isolated from wild type mice using a BD FACS Aria II cell sorter. We then treated these two cell types with LPS for 96 h.

**Fig. 6:**
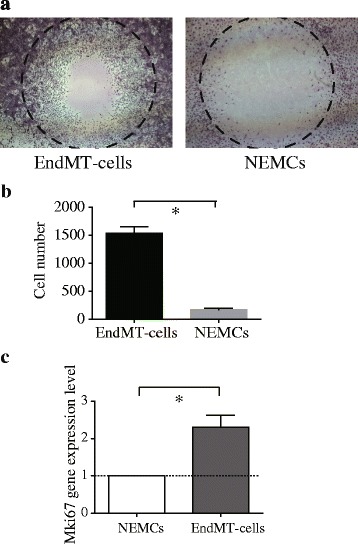
EndMT cells had increased proliferative activity relative to non-endothelium-derived mesenchymal cells. **a** Representative photos of wound healing cell migration assay. **b** Cells that migrated into the circle were measured in a wound healing assay. EndMT cells had significantly higher migration activity than non-endothelium-derived mesenchymal cells (*P < 0.05, *N* = 5). Values are means ± SEM. **c** Quantitative PCR analyses of EndMT cells and non-endothelium derived mesenchymal cells that were both treated with LPS for 96 h were performed to evaluate expression of Ki67, a proliferation marker. Ki67 mRNA levels were significantly upregulated in EndMT cells (*P < 0.05, *N* = 5). Values are means ± SEM

Similar to our previous report [3], almost all PVECs underwent a morphological change to acquire a spindle-like shape, and we defined these cells as EndMT-PVECs. A comparison of EndMT-PVECs and non-endothelium-derived mesenchymal cells (NEMCs) (CD31^−^/CD45^−^/CD326^−^ cells treated with LPS for 96 h) in a cell migration assay showed that EndMT-cells had higher migration activity compared to non-endothelium-derived mesenchymal cells (Fig. 6a, b). Quantitative PCR analyses of expression of the proliferation marker Ki67 by EndMT-cells and NEMCs treated with LPS for 96 h showed significant upregulation of Ki67 mRNA levels in EndMT-cells (*P < 0.05, *N* = 5) (Fig. 6c).

## Discussion

In this study, we first demonstrated that pulmonary vascular EndMT occurred in a murine model of pulmonary fibrosis after systemic endotoxemic injury. Although we recently reported that single LPS administration to mice was associated with transient pulmonary vascular EndMT without pulmonary fibrosis, whether intermittent LPS stimulation could also promote pulmonary fibrosis via EndMT was unclear [3]. Moreover, we also found that DPP-4 expression was increased in PVECs from a post-ALI pulmonary fibrosis murine model. Here we showed that treatment with the DPP-4 inhibitor vildagliptin effectively suppressed EndMT and had an anti-fibrotic effect in post-ALI pulmonary fibrosis.

Sepsis is the leading cause of ALI, which is characterized by endothelial activation and damage [25–27]. Although fibroblasts were long believed to originate directly from embryonic mesenchymal cells [28–30], endothelial cells are now seen as a source for fibroblasts in many tissues [7, 28, 31, 32]. The results of the present study demonstrate that persistent systemic endotoxemic injury also leads to pulmonary fibrosis via EndMT. Interestingly, interactions between LPS to PVECs could be in part direct and do not require immune cell mediation. These results are consistent with the clinical findings that acute lung injury develops in the presence of severe neutropenia.

Considering these results together with those shown in our recent report [3], pulmonary vascular EndMT is reversible and does not contribute to pulmonary fibrosis when endotoxemia is transient, but could lead to pulmonary fibrosis when endotoxemia persists. Although we have not determined whether pulmonary vascular EndMT is reversible in the post-ALI pulmonary fibrosis murine model, we did find that pulmonary fibrosis scores failed to improve 42 days after the initial administration of LPS (data not shown).

On the other hand, endotoxemia induces upregulation of DPP-4 expression in vascular endothelial cells, and DPP-4 inhibitors are reported to act as vascular endothelial protectors [19, 33, 34]. Examination of the role of DPP-4 in pulmonary disease was previously limited to respiratory epithelial injury such as bronchial asthma [11]. Although DPP-4 was recently reported to be involved in pathologic features of asthmatic airway inflammation, cell proliferation, and fibronectin production [35], there are few studies concerning the role of DPP-4 in pulmonary vascular function. Thus, we hypothesized that the DPP-4 inhibitor vildagliptin could attenuate pulmonary fibrosis by inhibiting EndMT.

Our in vivo and in vitro results show that in our model of pulmonary fibrosis after systemic endotoxemic injury, DPP-4 expression is upregulated in PVECs in both the presence and absence of immune cells. Vildagliptin treatment attenuated the accumulation of DPP-4 in PVECs, and was associated with an inhibition of fibrotic change and reduced EndMT-cells in lungs. Although other means of DPP-4 inhibition that do not involve endothelial cells could have promoted the observed anti-fibrotic effects, we strongly believe in the clinical significance of endothelial DPP-4 in fibrotic disorders such as pulmonary fibrosis after systemic endotoxemic injury, in which vascular damage and activation of endothelial cells play a significant role. Indeed, our study showed that EndMT cells had higher proliferation and migration rates compared to non-endothelium-derived mesenchymal cells. These results might suggest the clinical significance of suppressing the activity of EndMT-cells in post-ALI pulmonary fibrosis via a DPP-4 pathway.

Another novelty of this study is to show a direct action of DPP-4 inhibitors on ROS production in PVECs and attenuating EndMT. Since Yan et al. demonstrated that GLP-1 treatment could protect against the hyperglycemia-induced EndMT [36], we expected in this study that the EndMT-inhibiting effect was mediated by GLP-1. Although that would be true, our cellular model confirmed that DPP-4 inhibitors could attenuate EndMT even in the absence of GLP-1. For investigating the mechanistic insight, we evaluated if DPP-4 inhibitors attenuate ROS production in PVECs, which was reported to be the key trigger of EndMT in LPS-induced lung injury [3]. Interestingly, DPP-4 inhibitors directly induced antioxidant-response in PVECs. Taken together with our recent report, vildagliptin has been proven to protect against EndMT partly via suppressing ROS in PVECs, and it is partly independent of GLP-1.

Although oral DPP-4 inhibitors are used to treat diabetes [37], intraperitoneal injection of vildagliptin did not lead to significant hypoglycemia in this study (data not shown). This is consistent with earlier findings that DPP-4 inhibitors do not increase hypoglycemia rates, even though they increase insulin secretion in a glucose-dependent manner [38]. In the presence of sepsis or septic lung injury, we encounter many patients who suffer from hyperglycemia due to increased insulin resistance [39]. The target range for blood glucose in these conditions is the subject of considerable debate, but the lower end of the blood glucose range is not recommended for critically ill adults [40]. There is also a significant relationship between acute glucose swings and activation of oxidative stress [41], which might lead to an increase in vascular endothelial cell injury and organ dysfunction. Vildagliptin has shown the ability to prevent pulmonary fibrosis and did not induce severe hypoglycemia in this study indicating it may be effective for systemic endotoxemic lung injury.

There are several limitations of this study that should be considered. First, we have not examined whether other DPP-4 inhibitors could be beneficial for inhibiting post-ALI pulmonary fibrosis in vivo. We chose vildagliptin for in vivo experiments since it is the only DPP-4 inhibitor which has previously been administered intraperitoneally to the best of our knowledge [42]. An intermittent LPS-injected model revealed prominent oral intake loss for the first ten days, and their oral intake amount was unstable. Therefore, drugs which require oral administration are less reliable for these experiments, driving our decision to use vildagliptin. Shi et al. did report different activities in endothelial cells among various DPP-4 inhibitors [43], indicating that additional studies should focus on other DPP-4 inhibitors to determine whether these molecules have unique drug-specific effects. Second, we have only established the efficacy of vildagliptin in a preventive protocol, but not in a therapeutic protocol. To determine whether this molecular approach may have therapeutic relevance, we will need to start delivering vildagliptin from 4 or 5 days after initial LPS administration.

## Conclusions

The current study demonstrated that in a pulmonary fibrosis murine model after systemic endotoxemic injury, EndMT was observed in endothelial cells that overexpressed DPP-4. Vildagliptin might play a beneficial role in ameliorating pulmonary fibrosis by inhibiting EndMT even in the absence of GLP-1.

## Additional file

Additional file 1: Vildagliptin ameliorates pulmonary fibrosis in lipopolysaccharide-induced lung injury by inhibiting endothelial-to-mesenchymal transition (PDF 449 kb)

## Abbreviations

ALI: Acute lung injury
ARDS: Acute respiratory distress syndrome
DCFDA: Dichlorofluorescein diacetate
DPP-4: Dipeptidyl peptidase-4
EBM-2: Endothelial cell basal medium-2
EGM-2: Endothelial cell growth medium 2
EndMT: Endothelial-to-mesenchymal transition
EVG: Elastica van Gieson
FCM: Flow cytometry
GLP-1: Glucagon-like peptide-1
ICC: Immunocytochemistry
IHC: Immunohistochemistry
LPS: Lipopolysaccharide
MEndT: Mesenchymal-to-endothelial transition
NEMCs: Non-endothelium-derived mesenchymal cells
PVECs: Pulmonary vascular endothelial cells
ROS: Reactive oxygen species
TGFβ: Transforming growth factor β
