# Supplementary material for: Vildagliptin ameliorates pulmonary fibrosis in lipopolysaccharide-induced lung injury by inhibiting endothelial-to-mesenchymal transition
Source: Respir Res. 2017 Oct 16;18:177. doi: 10.1186/s12931-017-0660-4 (PMC5644255; doi:10.1186/s12931-017-0660-4)
Supplement: Additional file 1: — Vildagliptin ameliorates pulmonary fibrosis in lipopolysaccharide-induced lung injury by inhibiting endothelial-to-mesenchymal transition (PDF 449 kb) [file 12931_2017_660_MOESM1_ESM.pdf]

Online supplementary data

Title：Vildagliptin ameliorates pulmonary fibrosis in lipopolysaccharide-induced lung injury by inhibiting endothelial-to-mesenchymal transition

Authors：Toshio Suzuki, et al.

Supplementary methods

Sequences of the qRT-PCR primers

Pecam1: forward, 5'-AAGCCAACAGCCATTACGGT-3', reverse, 5'-AGCCTTCCGTTCTCTTGGTG3';
Cdh5: forward, 5'-GTTCAAGTTTGCCCTGAAGAA-3', reverse, 5'-GTGATGTTGGCGGTGTTGT-3';
Vwf: forward, 5'-CCAAGGAGGGTCTGCAACT-3', reverse, 5'-AAAGGAAGACTCTGGCAAGCTA-3';
Nos3: forward, 5'-ATCCAGTGCCCTGCTTCA-3', reverse, 5'-GCAGGGCAAGTTAGGATCAG-3';
Col1a1: forward, 5'-CATGTTCAGCTTTGTGGACCT-3', reverse, 5'-GCAGCTGACTTCAGGGATGT-3';
Col1a2: forward, 5'-GCAGGTTCACCTACTCTGTCCT-3', reverse, 5'-CTTGCCCCATTCATTTGTCT-3';
S100a4: forward, 5'-CACAGGAAACTTCGCAGTGA-3', reverse, 5'-CCTTCTGGAAGGAACAAATGA-3';
Acta2: forward, 5'-CTCTCTTCCAGCCATCTTTCAT-3', reverse, 5'-TATAGGTGGTTTCGTGGATGC-3';
Myh11: forward, 5'-CACAGGAAACTTCGCAGTGA-3', reverse, 5'-CCTTCTGGAAGGAACAAATGA-3';
Snai1: forward, 5'-CTTGTGTCTGCACGACCTGT-3', reverse, 5'-CAGGAGAATGGCTTCTCACC-3';
Snai2: forward, 5'-CATTGCCTTGTGTCTGCAAG-3', reverse, 5'-AGAAAGGCTTTTCCCCAGTG-3';
Twist1: forward, 5'-AGCTACGCCTTCTCCGTCT-3', reverse, 5'-TCCTTCTCTGGAAACAATGACA-3';
Twist2: forward, 5'-CATGTCCGCCTCCCACTA-3', reverse, 5'-GATGTGCAGGTGGGTCCT-3';
Zeb1: forward, 5'-AGGTGATCCAGCCAAACG-3', reverse, 5'-GGTGGCGTGGAGTCAGAG-3';
Zeb2: forward, 5'-CCAGAGGAAACAAGGATTTCAG-3', reverse, 5'-AGGCCTGACATGTAGTCTTGTG-3';
Hprt1: forward, 5'-TCCTCCTCAGACCGCTTTT-3', reverse, 5'-CCTGGTTCATCATCGCTAATC-3';
